# The Prevalence of Nephrolithiasis and Associated Risk Factors Among the Population of the Riyadh Province, Saudi Arabia

**DOI:** 10.7759/cureus.55870

**Published:** 2024-03-09

**Authors:** Abdullah M Alshubaili, Abdulaziz F Alotaibi, Khalid A Alsaleh, Abdulaziz I Almogarri, Abdullah A Alanizi, Saif S Alsaif, Ahmad S Alghamdi, Nasser M Alnazari, Amwaj Almalki

**Affiliations:** 1 Department of Medicine, King Saud Bin Abdulaziz University for Health Sciences College of Medicine, Riyadh, SAU; 2 Department of Hepatobiliary Sciences and Organ Transplant, King Saud Bin Abdulaziz University for Health Sciences College of Medicine, Riyadh, SAU; 3 Biostatistics and Epidemiology, King Abdullah International Medical Research Center, Riyadh, SAU

**Keywords:** prevalence, nephrolithiasis, renal stones, saudi arabia, urolithiasis

## Abstract

Background and objective

Kidney stones, also referred to as nephrolithiasis or renal calculi, is a condition where crystal depositions are formed within the kidney and ideally excreted from the body via the urethra with no pain; however, larger calculi may cause significant pain and require further medical assistance. The vast majority of patients who develop renal calculi form calcium stones, which are either a composition of calcium oxalate or calcium phosphate. Other types include uric acid, struvite, and cysteine. While kidney stones are one of the most significant diseases among the Saudi population, which require an acute emergency intervention to prevent serious long-term complications, there are limited studies published regarding this condition in Saudi communities. In light of this, we performed this study to assess the prevalence, incidence, and risk factors of kidney stones among the population of Riyadh, Saudi Arabia.

Methods

This was a cross-sectional study conducted in Riyadh, Saudi Arabia between August and October 2023, aiming to estimate the prevalence and risk factors of nephrolithiasis among residents of the Riyadh province. Data were collected through an electronic questionnaire in both Arabic and English and distributed via social media in addition to barcode handouts in various selected venues in Riyadh. The questionnaire involved 12 questions categorized into three sections. The first section obtained demographical information while the second section collected data about the past medical history of the participants. Lastly, the third section aimed to assess the prevalence of nephrolithiasis among participants or any history of the condition among their families.

Results

A total of 1,043 participants were surveyed, of whom 533 were males (51.1%). The prevalence of kidney stones was reported in 98 individuals (9.4%) overall. Individuals in the age groups of 36-50, 51-60, and >60 years showed significantly more renal stone prevalence than those in younger age groups (p<0.001). The prevalence was found to be higher in participants who were smokers, diabetic, hypertensive, and those who suffered from inflammatory bowel disease (IBD), gout, chronic kidney disease (CKD), hyperthyroidism, and hyperparathyroidism. Participants who took calcium supplements or had a positive family history of renal stones were found to have a higher prevalence of renal stones as well. However, only hypertension, gout, and family history showed any statistical significance (p<0.05).

Conclusions

A direct correlation was observed between hypertension, gout, positive family history, and aging and an increased prevalence of kidney stones among the inhabitants of the Riyadh province. Therefore, we encourage the local authorities to raise awareness of kidney stones and their related risk factors among the general public. Moreover, further local studies need to be conducted to gain deeper insights into kidney stone prevalence, especially pertaining to associated comorbidities and the pattern of the disease itself.

## Introduction

Kidney stones, also known as nephrolithiasis or renal calculi, is a condition characterized by the formation of crystal depositions within the kidney, which are ideally excreted from the body via the urethra without causing any pain; however, larger calculi may cause significant pain and necessitate medical treatment [1]. The vast majority of patients who develop renal calculi form calcium stones (80%). Furthermore, calcium stones are either a composition of calcium oxalate or calcium phosphate [1]. Other types of kidney stones include uric acid, struvite, and cysteine stones [1]. Stones form within the kidney in the setting of urine supersaturation, during which solutes begin to precipitate in the urinary tract, leading to crystal formation [2]. In addition, many other factors such as the pH level of the urine affect the formation of crystals within the kidney [2]. Patients with nephrolithiasis may not report any symptoms early in the course of the disease; however, later on, they may present with flank pain, hematuria, urinary tract infection (UTI), blockage of urine flow, obstructive uropathy, and hydronephrosis [1].

A study from the United States involving 10,521 participants and focusing on the prevalence and incidence of kidney stones reported a prevalence rate of 1,157 (11%) and a 12-month incidence rate of 2.1% (n=221) [3]. Moreover, another US study assessing the prevalence of kidney stones among 12,110 participants reported them in 1,066 (8.8%) participants [4]. Additionally, the study found that kidney stone prevalence was higher in obese men compared to other participants [4]. Locally, in Saudi Arabia, a retrospective study assessing the characteristics and types of kidney stones in the Eastern Region found that among 235 reviewed patients with a mean age of 48.52 years, 175 (74.5%) had renal calculi, with calcium oxalate being the most common type (n=133, 76%) [5]. The study reported a male predominance in the patient population. 

Risk factors related to kidney stones vary among subsets of populations, and environmental factors play a crucial role in the pathogenesis of the condition. Furthermore, extensive research has revealed that the incidence of nephrolithiasis can be correlated with gender, ethnicity, geographical location, occupation, hot climate, and an unhealthy diet, such as those involving excessive consumption of caffeine, salt, dairy products, animal proteins, and fat [6]. In addition, individuals with a prior history of kidney stones are at a higher risk of developing new kidney stones [7] by 15% during the first year, and 50% in the next 10 years [8]. Also, low fluid intake is highly associated with the incidence and recurrence of kidney stones [9], with studies showing that high fluid intake minimizes the risk of developing kidney stones [9]. Comorbidities such as metabolic syndrome have been strongly linked to the formation of kidney stones [10]. Thus, for instance, a patient who has three or more metabolic syndrome traits is more prone to develop kidney stones [10].

Kidney stones are one of the most significant diseases among the Saudi population, which require an acute emergency intervention to prevent serious long-term complications; however, there are limited studies published regarding this disease in the Saudi population. Hence, this study aimed to examine the prevalence, incidence, and risk factors of kidney stones among the population of Riyadh, Saudi Arabia.

## Materials and methods

Study design and setting

This was a cross-sectional study conducted in Riyadh, Saudi Arabia after obtaining IRB approval from the King Abdullah International Medical Center (approval no. IRB-1878-23). The study was conducted between August 2023 and October 2023. Data were collected through an electronic questionnaire in both Arabic and English languages distributed via social media in addition to barcode handouts in various selected venues in Riyadh. Furthermore, information was kept private per Google’s privacy policy. Participation was strictly voluntary.

Sample size

The sample size was estimated to be 385 with a confidence interval of 95% and a margin of error of 5% based on calculations performed on the Raosoft online calculator (Raosoft, Inc., Seattle, WA) where the population of Riyadh province was estimated to be 8.5 million according to data extracted from General Authority for Statistics (GASTAT) [11]. The inclusion criteria were any resident in Riyadh province aged 18 years or more. Individuals not residing in the Riyadh province and those aged less than 18 years were excluded from the study. A total of 1208 respondents completed the survey, of which 164 were excluded for not meeting the inclusion criteria.

Development and application of the questionnaire

The research team contacted the corresponding author of a study that was conducted in Hail City about obtaining and using the questionnaire in our research [12]. The questionnaire involved 12 questions categorized into three sections. The first section obtained demographical information such as age, gender, height, weight, education level, and occupation. The second section collected the past medical history of the participants, including chronic diseases, use of medication, and whether they smoked or not. The third section assessed the prevalence of nephrolithiasis among participants or any pertinent history among their families.

Data management and statistical analysis

Data collected were entered into a Microsoft Excel sheet and tabulated. Data analysis was performed on SPSS Statistics version 23 (IBM Corp., Armonk, NY) by an independent biostatistician. Descriptive statistics were presented in the form of frequencies and percentages. Mean and standard deviation (SD) were used for representing the continuous variables. Pearson’s chi-square test was used to assess the relationship between categorical variables. A multivariate regression model was performed to analyze the risk factors for renal stones. A p-value less than 0.05 was considered statistically significant.

## Results

Table 1 presents the sociodemographic characteristics of the surveyed population (n=1,043). Gender distribution was almost evenly split, with 510 (48.9%) female and 533 (51.1%) male respondents, but age distribution showed a varying pattern. The majority were in the age groups of 18-25 and 36-50 years. A total of 407 (39.0%) were in the 18-25-years age group and 249 (23.9%) were aged 36-50 years, while individuals aged over 60 constituted the smallest segment with 60 participants (6%). As for nationality, the participants were predominantly Saudi (n=1,002, 96.1%). A significant majority of the subjects had an education of university level or above (n=746, 71.5%). Occupationwise, non-healthcare workers formed the largest group, comprising 793 (76.0%) participants, followed by healthcare workers (n=153, 14.7%) and unemployed individuals (n=97, 9.3%). Regarding BMI distribution, 397 (38.1%) individuals were overweight, 358 (34.3%) were within the normal weight range, 243 (23.3%) were obese, and only 45 (4.3%) were underweight, representing the most minuscule fraction of the sample.

**Table 1:** Sociodemographic characteristics of the study population BMI: body mass index

Sociodemographic characteristics			
		N	%
Gender	Female	510	48.9
	Male	533	51.1
Age group, years	18-25	407	39.0
	26-35	178	17.1
	36-50	249	23.9
	51-60	146	14.0
	>60	63	6.0
Nationality	Non-Saudi	41	3.9
	Saudi	1002	96.1
Educational level	Primary or lower	10	1.0
	Intermediate or secondary	179	17.2
	Diploma	108	10.4
	University or above	746	71.5
Occupation	Healthcare worker	153	14.7
	Non-healthcare worker	793	76.0
	Unemployed	97	9.3
BMI	Underweight	45	4.3
	Normal	358	34.3
	Overweight	397	38.1
	Obese	243	23.3

**Figure 1:**
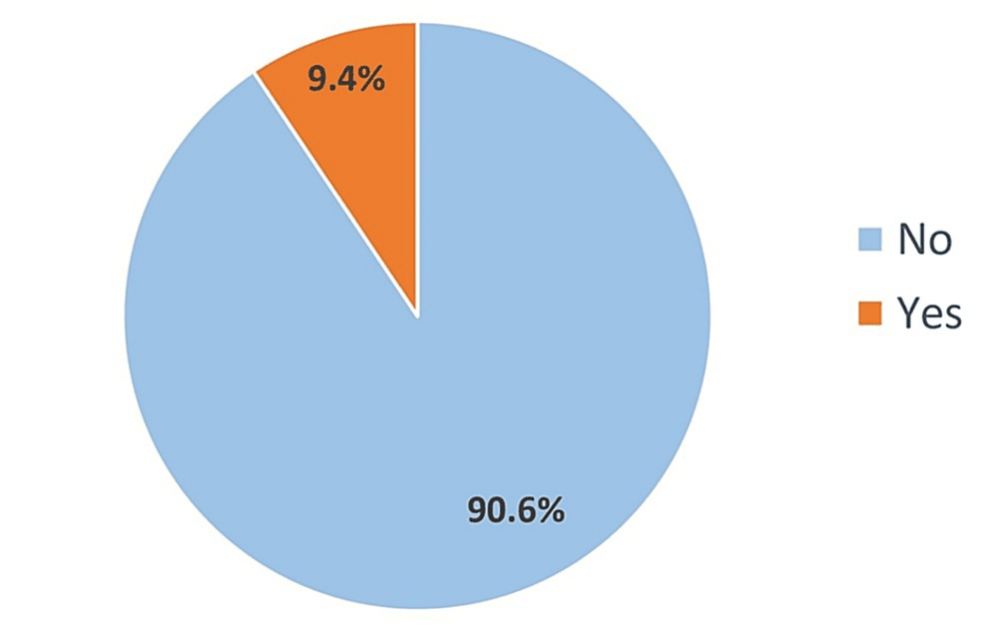
Prevalence of renal stones

Renal stones were reported in 98 participants (9.4%), as illustrated in Figure 1.

The medical and personal histories of the participants were assessed, and smoking was reported by 198 (19%) respondents. Prevalence rates for specific medical conditions varied, with inflammatory bowel disease (IBD) among 28 (2.7%) participants, hypertension in 97 (9.3%), diabetes mellitus in 84 (8.1%), gout in 21 (2.0%), chronic kidney disease (CKD) in seven (0.7%), hyperthyroidism in 16 (1.5%), hypothyroidism in 19 (1.8%), and hyperparathyroidism in four (0.4%). Medication usage was reported by 539 (52.7%) participants, with 414 (39.7%) using vitamin D supplements, 98 (9.4%) using calcium supplements, and 27 (2.6%) using diuretics; 380 (36.4%) reported a family history of renal stones.

The prevalence of renal stones based on different sociodemographic characteristics is presented in Table 2. No significant differences were seen between male and female participants regarding renal stone prevalence (p=0.209). The age groups of 36-50, 51-60, and >60 years showed significantly more renal stone prevalence than younger age groups (p<0.001). Nationality, educational level, occupation, and BMI did not show statistically significant differences regarding renal stone prevalence (p>0.05).

**Table 2:** Prevalence of renal stones based on the sociodemographic characteristics of the participants *Statistically significant (p<0.05) BMI: body mass index

Prevalence of renal stones based on sociodemographic characteristics							
			Renal stone				
			No	Yes	Total	P-value	Chi-square value
Gender	Female	N	468	42	510	0.209	1.579
		%	91.8%	8.2%	100.0%		
	Male	N	477	56	533		
		%	89.5%	10.5%	100.0%		
Age group, years	18-25	N	395	12	407	<0.001^*^	45.594
		%	97.1%	2.9%	100.0%		
	26-35	N	166	12	178		
		%	93.3%	6.7%	100.0%		
	36-50	N	214	35	249		
		%	85.9%	14.1%	100.0%		
	51-60	N	117	29	146		
		%	80.1%	19.9%	100.0%		
	>60	N	53	10	63		
		%	84.1%	15.9%	100.0%		
Nationality	Non-Saudi	N	36	5	41	0.531	0.393
		%	87.8%	12.2%	100.0%		
	Saudi	N	909	93	1002		
		%	93.3%	6.7%	100.0%		
Educational level	Primary or lower	N	9	1	10	0.591	1.909
		%	90.0%	10.0%	100.0%		
	Intermediate or secondary	N	167	12	179		
		%	93.3%	6.7%	100.0%		
	Diploma	N	98	10	108		
		%	90.7%	9.3%	100.0%		
	University or above	N	671	75	746		
		%	89.9%	10.1%	100.0%		
Occupation	Healthcare worker	N	144	9	153	0.224	2.993
		%	94.1%	5.9%	100.0%		
	Non-healthcare worker	N	712	81	793		
		%	89.8%	10.2%	100.0%		
	Unemployed	N	89	8	97		
		%	91.8%	8.2%	100.0%		
BMI	Underweight	N	43	2	45	0.073	6.962
		%	95.6%	4.4%	100.0%		
	Normal	N	334	24	358		
		%	93.3%	6.7%	100.0%		
	Overweight	N	353	44	397		
		%	88.9%	11.1%	100.0%		
	Obese	N	215	28	243		
		%	88.5%	11.5%	100.0%		

The prevalence of renal stones was found to be higher in participants who were smokers; those with IBD, hypertension, diabetes mellitus, gout, CKD, hyperthyroidism, and hyperparathyroidism; those who took calcium supplements; and those with a family history of renal stones. However, only hypertension, gout, and family history showed statistical significance (p<0.05) (Table 3).

**Table 3:** Prevalence of renal stones based on the medical and personal history of the participants *Statistically significant (p<0.05)

Prevalence of renal stones based on medical and personal history							
			Renal stone				Chi-square value
			No	Yes	Total	P-value	
Smoking	No	N	771	74	845	0.144	2.132
		%	91.2%	8.8%	100.0%		
	Yes	N	174	24	198		
		%	87.9%	12.1%	100.0%		
Inflammatory bowel disease (IBD)	No	N	921	94	1,015	0.369	0.808
		%	90.7%	9.3%	100.0%		
	Yes	N	24	4	28		
		%	85.7%	14.3%	100.0%		
Hypertension	No	N	868	78	946	<0.001^*^	15.882
		%	91.8%	8.2%	100.0%		
	Yes	N	77	20	97		
		%	79.4%	20.6%	100.0%		
Diabetes mellitus	No	N	872	87	959	0.226	1.469
		%	90.9%	9.1%	100.0%		
	Yes	N	73	11	84		
		%	86.9%	13.1%	100.0%		
Gout	No	N	930	92	1,022	0.002^*^	0.257
		%	91.0%	9.0%	100.0%		
	Yes	N	15	6	21		
		%	71.4%	28.6%	100.0%		
Chronic kidney disease (CKD)	No	N	939	97	1,036	0.656	0.198
		%	90.6%	9.4%	100.0%		
	Yes	N	6	1	7		
		%	85.7%	14.3%	100.0%		
Hyperthyroidism	No	N	932	95	1,027	0.196	1.670
		%	90.7%	9.3%	100.0%		
	Yes	N	13	3	16		
		%	81.3%	18.8%	100.0%		
Hypothyroidism	No	N	927	97	1,024	0.656	0.388
		%	90.5%	9.5%	100.0%		
	Yes	N	18	1	19		
		%	94.7%	5.3%	100.0%		
Hyperparathyroidism	No	N	942	97	1,039	0.284	1.148
		%	90.7%	9.3%	100.0%		
	Yes	N	3	1	4		
		%	75.0%	25.0%	100.0%		
Vitamin D supplements	No	N	569	60	629	0.845	0.038
		%	90.5%	9.5%	100.0%		
	Yes	N	376	38	414		
		%	90.8%	9.2%	100.0%		
Calcium Tablets	No	N	860	85	945	0.168	1.902
		%	91.0%	9.0%	100.0%		
	Yes	N	85	13	98		
		%	86.7%	13.3%	100.0%		
Diuretics	No	N	918	98	1,016	0.090	2.874
		%	90.4%	9.6%	100.0%		
	Yes	N	27	0	27		
		%	100.0%	0.0%	100.0%		
Family history of renal stone	No	N	612	51	663	0.013^*^	6.204
		%	92.3%	7.7%	100.0%		
	Yes	N	333	47	380		
		%	87.6%	12.4%	100.0%		

A binary logistic regression analysis was performed to assess the risk factors of renal stones (Table 4). BMI >30 kg/m^2^ [OR=1.59 (95% CI: 0.98-2.59), p=0.049], educational level of university or higher [OR=1.97 (95% CI: 1.03-3.77), p=0.042], hypertension [OR=3.02 (95% CI: 1.58- 5.78), p=0.001], gout [OR=3.33 (95% CI: 1.09-10.14), p=0.035], and family history of renal stones [OR=1.73 (95% CI: 1.11-2.71), p=0.016] were found to be independently associated with renal stones.

**Table 4:** Logistic regression model to assess the risk factors for renal stones *Statistically significant (p<0.05) CI: confidence interval; BMI: body mass index; SE: standard error; df: degrees of freedom

Logistic regression model						
Variables	B	SE	Wald	df	Odds ratio (95% CI)	P-value
Male gender	0.222	0.254	0.763	1	1.25 (0.76 -2.05)	0.383
Age =>50 years	0.486	0.434	1.252	1	1.63 (0.69-3.81)	0.263
BMI =>30 kg/m^2^	0.466	0.249	3.506	1	1.59 (0.98-2.59)	0.049^*^
Saudi nationality	-0.251	0.521	0.232	1	0.78 (0.28- 2.16)	0.630
Educational level of university or higher	0.676	0.332	4.142	1	1.97 (1.03-3.77)	0.042^*^
Non-healthcare occupation	0.434	0.371	1.367	1	1.54 (0.75-3.19)	0.242
Smoking	0.294	0.282	1.081	1	1.34 (0.77-2.33)	0.298
Inflammatory bowel disease (IBD)	0.216	0.586	0.136	1	1.24 (0.39-3.91)	0.712
Hypertension	1.106	0.331	11.186	1	3.02 (1.58- 5.78)	0.001^*^
Diabetes	0.118	0.419	0.080	1	1.13 (0.50-2.56)	0.778
Gout	1.202	0.569	4.468	1	3.33 (1.09-10.14)	0.035^*^
Chronic kidney disease (CKD)	-1.011	1.228	0.678	1	0.36 (0.03-4.04)	0.410
Hyperthyroidism	0.399	0.737	0.293	1	1.49 (0.35-6.31)	0.588
Hypothyroidism	-0.633	1.053	0.361	1	0.53 (0.07-4.18)	0.548
Hyperparathyroidism	1.956	1.247	2.462	1	7.07 (0.61-81.40)	0.117
Take vitamin D	-0.155	0.247	0.392	1	0.86 (0.53- 1.39)	0.531
Take calcium	0.290	0.368	0.619	1	1.34 (0.65-2.75)	0.431
Take diuretics	-20.094	7299.287	0.000	1	0.00 (0.00-0.00)	0.998
Family history of renal stones	0.548	0.228	5.787	1	1.73 (1.11-2.71)	0.016^*^
Constant	-3.869	0.712	29.493	1	0.021	0.000

## Discussion

Kidney stones remain one of the most common conditions encountered in acute care settings [13]. Recent studies have shown an increase in the prevalence of kidney stones globally [14]. Similarly, recent research done in Saudi Arabia to estimate the prevalence of kidney stones in Jeddah and Riyadh has shown that the disease prevalence is on the rise [15]. Therefore, gaining deeper insights into this topic is important not only for treatment guidance but also for identifying the associated risk factors and further modifying them accordingly. Moreover, an assessment of the current prevalence of this disease in our region is pivotal as it reflects the burden on the community, quality of life, and financial costs. In this study, renal stones were reported in 98 participants (9.4%) in the Riyadh province. Another study conducted in Saudi Arabia reported renal stones among 64 patients (9.1%), which is slightly lower than in our study [16]. Another study from Riyadh and Jeddah found that the prevalence in Riyadh city alone was 14.8% (n=56), which is higher than the outcome observed in our study; however, this might be attributed to the lower sample size in the study from Riyadh province [15].

Regarding age, the highest occurrence of kidney stones was among those aged 36 and older, and the prevalence was noted to increase with advancing age. These findings are similar to another study conducted in Bisha, which found that those aged 51 and older are more likely to have kidney stones [17]. These findings can be attributed to the continuous defects in urine ammoniagenesis, which is considered the main factor behind low urine pH that leads to the formation of kidney stones [18]. Concerning gender, our study did not show any statistically significant difference. However, the results revealed a slightly higher male predominance (n=533, 10.5%) when compared to females (n=510, 8.2%). These findings align with another study from Saudi Arabia [16]. However, another study found that the male predominance was much higher when compared to our results [5]. These observations can be linked to the role of estrogen, which plays a protective role in decreasing the concentration of urinary calcium and calcium oxalate [19]. On the other hand, a study done by Safdar et al. and Bokhari et al. showed that the female gender is at a higher risk of developing kidney stones [12,15]. Furthermore, recent studies have pointed towards a noticeable narrowing in the gap between the gender ratio [20].

As for BMI, a BMI of over 30 kg/m^2^ ​​​​​​was found to be independently associated with urolithiasis in this study. Furthermore, a study that evaluated the relationship between BMI and the risk of kidney stone formation found a significant trend between high BMI and the formation of kidney stones [21]. These findings can be explained by the role of insulin resistance in patients with a higher BMI. Subsequently, insulin resistance leads to a decrease in urinary pH, which in turn influences the formation of uric acid stones [21]. With regard to smoking, this study showed that the prevalence of kidney stones is higher in smokers. This is consistent with a meta-analysis published in 2023, which showed that smokers were at almost nine times higher risk of developing kidney stones when compared to those who had never smoked [22]. This can be attributed to the increase in vasopressin levels, which can lead to urinary retention, further contributing to a higher risk of stone formation [22].

Concerning diabetes, our study found that the prevalence of nephrolithiasis was higher among diabetic patients; however, diabetes as an independent risk factor did not show any statistical significance regarding stone formation. Nevertheless, a meta-analysis conducted in 2015 involving seven studies showed that diabetes correlates with a significant increase in the risk of kidney stone formation compared to those without the disease [23]. The study further reported that BMI, hypertension, and cigarette smoking are factors that affect the association between diabetes and kidney stone formation [23]. Moreover, diabetes is significantly associated with uric acid stones; this can be explained by the fact that diabetics have increased insulin resistance, leading to a lower urine pH [23].

Regarding comorbidities, this study showed a strong correlation between hypertension and gout and the incidence of urolithiasis. These results are in line with another study done in Japan to assess the prevalence of kidney stones in patients with gout [24]. Furthermore, another study focusing on the population of renal stone patients found a positive association between hypertension and the formation of renal stones [25]. A possible hypothesis for this correlation is hypercalciuria, given that most hypertensive patients have an increased urinary calcium excretion, which leads to the formation of calcium-containing stones such as calcium oxalate and calcium phosphate [26]. Regarding gout patients, the predisposition toward renal stones could be attributed to low urinary pH and decreased fractional excretion of uric acid [27].

Other parameters in our study, such as the use of vitamin D, calcium tablets, and diuretics, were not significantly associated with urolithiasis. In general, diuretics play a protective role in the formation of renal stones regardless of whether loop or thiazide diuretics are used [28]. Concerning family history, this study found that a positive family history of kidney stones increases the risk of developing nephrolithiasis. These observations align with other studies that investigated the relationship between family history and the risk of developing kidney stones, which have found that a positive family history increases the risk of kidney stone formation [29]. This can be explained by several genetic factors that are believed to contribute to the formation of calcium oxalate stones and consequently lead to abnormal excretion of calcium oxalate, citrate, and uric acid promotors or suppressors [29].

As for occupation, this study compared healthcare workers with non-healthcare workers concerning renal stone prevalence. We concluded that there is no significant difference in the prevalence of renal stones between healthcare workers and non-healthcare workers. In contrast, another study conducted in 2016 found that physicians had lower rates of nephrolithiasis than the general population and other healthcare workers [30]. The same study found that pharmacists, nurses, and other healthcare workers showed no significant difference regarding this condition when compared to the general population [30]. Therefore, we can establish that these discrepancies are due to certain limitations in our study, such as the failure to specify the precise role of healthcare workers, and may also be attributed to the difference in sample size between the two populations.

Limitations

Since the survey was distributed via social media, most respondents were from the younger age groups, and this may have affected the outcomes in terms of the true prevalence of the disease in the community. Additionally, we relied on self-reporting for data collection, which also may have affected the results regarding the actual prevalence of kidney stones. Moreover, our sample size was insufficient to assess the potential effects of comorbidities such as IBD, CKD, hypothyroidism, hyperthyroidism, and hyperparathyroidism.

## Conclusions

This study revealed that the prevalence of kidney stones among the residents of Riyadh province is high, with a clear association with increasing age, positive family history, hypertension, and gout. We urge local authorities to spread awareness about kidney stones and their related risk factors. Moreover, more local studies with larger sample sizes need to be conducted to determine the prevalence of kidney stones in Saudi Arabia on a broader scale, especially by factoring in other elements such as associated comorbidities and the pattern of the disease itself.
